# Inflammation blood and tissue factors of plaque growth in an experimental model evidenced by a systems approach

**DOI:** 10.3389/fgene.2014.00070

**Published:** 2014-04-07

**Authors:** Gualtiero Pelosi, Silvia Rocchiccioli, Antonella Cecchettini, Federica Viglione, Mariarita Puntoni, Oberdan Parodi, Enrico Capobianco, Maria G. Trivella

**Affiliations:** ^1^Institute of Clinical Physiology, Consiglio Nazionale delle RicerchePisa, Italy; ^2^Department of Clinical and Experimental Medicine, University of PisaPisa, Italy; ^3^Laboratory of Integrative Systems Medicine, Institute of Clinical Physiology, Consiglio Nazionale delle RicerchePisa, Italy; ^4^Center for Computational Science, University of MiamiMiami, FL, USA

**Keywords:** systems biomedicine, coronary atherogenesis, swine model, vascular inflammation, Bayesian model averaging

## Abstract

**Purpose:** The multifactorial pathogenesis of coronary atherosclerotic lesion formation has been investigated in a swine model of high cholesterol diet induced atherogenesis and data processed by a systems approach.

**Methods:** Farm pigs were fed on standard or high cholesterol diet of 8 and 16 weeks duration. Plasma assessment of total cholesterol, HDL, LDL, and ELISA of some cytokines and ICAM-1 were performed on baseline and end-diet samples. Segments of the right coronary artery were incubated for 24 h in serum-free medium to collect secreted proteins and their expression analyzed by mass spectrometry. Data of plasma and tissue factors were processed by a statistical systems inference approach: both histologic parameters of coronary intimal thickness (IT) and of lesion area (LA) were chosen as dependent variables (coronary atherosclerotic burden).

**Results:** Relations among plasma adhesion molecules, cytokines, lipoproteins, tissue proteins and histology indexes were integrated in a model regression scheme. Bayesian model averaging (BMA) variable selection was chosen as a method to identify relevant factors associated to atherosclerotic burden: TNFα was identified as an associated plasma marker, oxLDL and HDL as relevant lipoproteins; macrophage function related antioxidant Catalase enzyme, lysosome associated Cathepsin D, S100-A10, and Transforming growth factor-beta-induced protein ig-h3 were identified and selected as associated to atherogenesis outcome.

**Conclusions:** The results of this systems approach are consistent with the hypothesis that, in high cholesterol diet-induced experimental atherogenesis, the interaction between plasma cytokines, lipoproteins and artery-specific proteins, influences lesion initiation and growth. In particular, some macrophage function related proteins are found significantly and positively associated to atherosclerotic burden, suggesting a novel molecular framework into the atherogenesis-inflammatory disorder.

## Introduction

Atherogenesis is the initiating step of atherosclerosis, and can be considered the key-point for a better understanding of the entire process, as several factors and mechanisms are also related to plaque progression in the clinical scenario (Weber and Noels, 2011).

Plaque initiation steps take place in the following environments:

1. Systemic blood environment (proatherogenic or atheroprotective) constituted of inflammatory and lipid factors
2. Endothelial blood-vessel interface, which expresses adhesion molecules for monocyte intra-lesional transfer
3. Sub-endothelial intimal space, where proteoglycans retain LDL
4. Intimal and intima-media interface, scenario for vascular smooth cell (VSMC) phenotype switch and activation toward migratory and proliferative conditions (Libby et al., 2010)

Traditional views of atherosclerosis, basically seen as a lipid-based disorder, have been modified by the recognition of the multifactorial etiology of this disease (Lamon and Hajjar, 2008), involving the interplay of genetic, phenotypic and environmental factors that have to be integrated into a unified scheme. According to this theory, the most likely sequence of events occurring in the initial phase of atherosclerosis comprises vascular dysfunction and/or injury, monocyte recruitment and foam cell formation, lipid deposition, vascular smooth muscle cell proliferation and synthesis of extracellular matrix (Libby, 2002). The interaction of all these factors confers to the resulting atherosclerotic plaque its typical features.

In this study, circulatory systemic and locally expressed artery factors in a high cholesterol diet animal model of coronary atherogenesis have been collected and inter-related using a Bayesian Model Averaging (BMA) (Leamer, 1978; Raftery, 1995) computational approach, which is also suggested as a useful strategy to unravel novel actors and pathways outlining this complex framework.

A statistical regression framework based on BMA to account for model uncertainty determined by many variables of heterogeneous nature has been used. In such circumstances, the choice of an encompassing model is not easy, and needs to be a statistically reasonable decision. BMA is a suitable model strategy, which presents several advantages and reasonable computational requirements.

## Materials and methods

### Experimental design, circulatory-tissue data collection and histology

Animal experiment protocol was approved by the Animal Care Committee of the Minister of Public Health according with guidelines (protocol number: 06/2009-B-2009/01/26). Atherogenesis has been studied in 13 farm pigs fed on a high cholesterol (4%) high fat (27%) diet for 8 (HF, 4 cases) and 16 weeks (HHF, 6 cases) and controls fed on standard diet (CNTL, 3 cases). Data on plasma lipids, cytokines and cell adhesion markers have been collected before and at the end of the diet period in all animals. Total cholesterol, High Density Lipoprotein (HDL) and triglycerides (TG) were measured by standard enzymatic techniques (Synchron CX9 Pro, Beckman Coulter Inc., Fullerton, CA, USA). Low density lipoprotein (LDL) was calculated according to Friedewald et al. (1972) IL-6, TNFα, and ICAM-1 were purchased by Abcam (Cambridge, UK), while oxLDL was a product of Antibodies-Online (Atlanta, GA, USA).

At the end of diet period, animals were anesthetized by intramuscular administration of 10 mg/kg of Zoletil® and 0.05 mg/Kg of atropine, plus 5 mg/kg/h of propofol intravenous infusion and sacrificed by KCl i.v. bolus injection. Upon heart explantation, a 3 mm long segment of the proximal tract of right coronary artery (RCA), 1 cm below the ostium, was harvested and placed in serum free solution to collect secreted/released proteins (Rocchiccioli et al., 2013)

Following heart fixation in 10% buffered formalin (7–10 days), 5–10 mm thick transverse arterial samples were collected from left main, left anterior descending, left circumflex and right coronary arteries for routine histologic processing for paraffin embedding. Consecutive cross-sections were obtained from each coronary segment (rotary microtome Microm HM 300, Bio-optica) for Haematoxylin and Eosin, Mallory trichrome and Weigert van Gieson staining and examined under light microscopy (Olympus BX43, Italy) from 2× to 40× original magnification. Images were digitized by a video system (Olympus DP20 camera, Italy) interfaced to a computer with dedicated software (CellSens Dimension, Olympus, Italy) for morphometric analysis. Intimal thickness (IT, mm), i.e. maximal radial expansion of the lesion, and lesional area (LA, mm^2^) i.e., entire lesion area in each cross-section, were used as representative morphometric indexes of overall atherosclerotic burden in each individual case. Both mean and median of all the IT and LA values of all cross-sectioned coronary lesions of each case were calculated (Viglione et al., 2013).

### Liquid chromatography (LC) separation, mass spectrometry (MS) analyses and data post-processing

Chromatographic separation of digested peptides obtained from secreted proteins was performed using an Ultimate 3000 nano-HPLC system (LC Packings, DIONEX, USA) and peptides eluted from chromatography were directly processed using TripleTOF™ 5600 mass spectrometer (AB SCIEX, Toronto, Canada) (Rocchiccioli et al., 2013). MS/MS data were processed with ProteinPilot™ Software (AB SCIEX, Toronto, Canada), using the Paragon™ and Pro Group™ Algorithms and SwissProt 2012 as protein database for *Sus scrofa*. The false discovery rate (FDR) analysis was done using the integrated tools in ProteinPilot software and a confidence level of 95% was set. Expression data for proteins were obtained using MarkerView^TM^ software 1.2.1 (AB SCIEX). Normalization of the total artery tissue size was accomplished with a global normalization of profiles (total protein content) using Marker View 1.2 software.

### Mathematical model approach: implementation of R environment, BMA package

Circulatory and omics data have been processed and related to histology parameters of mean and median coronary IT and LA of each case of HF and HHF groups. All dependent and independent variables of diet treated cases (HF and HHF) were normalized to average values of standard diet CNTL cases which are taken as reference. The effect of normalization, together with a logarithmic transform taken to minimize variability, is a better control of the wide range of magnitude for the absolute values of histology, circulatory and omics variables. The independent variables are considered plasma lipoproteins (total cholesterol, LDL, HDL) oxidized LDL, circulatory cytokines (IL6 and TNFα, ICAM-1 and several coronary proteins identified by LC-MS reported in the Supplementary Table 1. At first, the model has been applied to all diet treated cases as a whole group, while it was subsequently applied to HF and HHF groups separately.

In general, BMA is employed when multiple models may be statistically reasonable, and selecting a single particular model can lead to the underestimation of the uncertainty related to the model form underlying the variables of interest. In such cases, BMA can quickly determine suitable models through specified sets of explanatory variables with high likelihoods. Equivalently, averaging across a large set of such models allows to determine the variables which are relevant to the data generating process for a given set of priors used in the analysis.

The implementation of BMA was done within the R environment (Raftery et al., 2010), by averaging the best models of a certain class, and according to the approximate posterior model probability which was computed in each case. For instance, the class “bicreg” in the BMA R package identifies the linear regression models, and is the one chosen among other possible tested classes. In this way the analysis has been kept at its simplest and most interpretable level. In particular, the option “iBMA” represents the iterated BMA method for variable selection, and works by repeatedly calling BMA, i.e., iterating through the variables in a fixed order based on some measure of goodness of fit. After each call, only the variables with posterior probability greater than a specified threshold are retained, the rest being replaced by other variables.

The summary function was used to provide concise and summarized information about the variables that have been examined up to the last iteration. Each model, and set of variables, is weighted and the final estimates are constructed as a weighted average of the parameter estimates from each of the models. All the variables are considered, but some are subject to shrinking by setting to zero the model weights, and depending upon features such as the choice of prior (see also Supplementary Material).

### Post-processing of model results

The adopted strategies to assess relevance of model selections were:

1. Congruence of model selection by histology indexes.

Among all selected variables, those with only IT or LA association have been discarded. Congruence of selected variables with both maximal radial expansion and circumferential extension of lesions was thus ensured.

2. Congruence of model selection by regression coefficients (value and sign).

It has also been checked whether relevant variables according to step 1 had regression coefficients of comparable size, and similar direction of association (negative or positive sign); this strategy allowed for a more robust combination of factors which strongly relate to atherogenesis outcome. Variables were discarded, when the corresponding coefficients had comparable size and opposite sign, indicating inappropriate selection, as well as when absolute values were very low (<0.001) irrespective of sign congruence.

## Results

Circulatory and omics data (Supplementary Table 1) have been processed by BMA and related to histology parameters of mean and median coronary IT (mm) and LA (mm^2^) of HF and HHF groups. Circulatory data were measured by antibody-based kits and expressed as a concentration in serum. Protein data were measured by mass spectrometry and protein expression was measured by peptide peak area using arbitrary units (normalized counts).

### Selected variables by first implementation run of the model: all high cholesterol diet-treated animals

IT and LA are the dependent variables that have been chosen for BMA approach to provide different and complementary information on atherosclerotic lesions, depending on lesion shape and its mainly eccentric or concentric growth. IT is more representative of maximal radial expansion of the lesion, whilst LA is more related to the circumferential extension.

**Figure 1:**
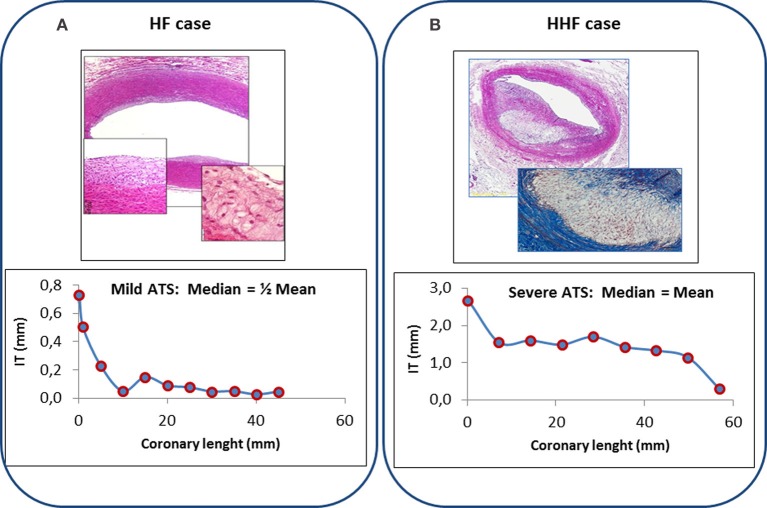
**Top: Histologic features of coronary lesions in a typical HF (panel A, fatty streak, H&E 2×, insets 10× and 20×) and HHF case (Panel B, atheroma, H&E 4×, inset Mallory trichrome 10×)**. Bottom: Coronary profiling (left anterior descending artery) of IT values of observed lesions in 11 consecutive segments of a HF case **(panel A)** and in 9 consecutive segments of a HHF case **(panel B)**. Median of IT values is about one half of the mean of IT values in mild localized atherosclerotic changes of HF case (left), whilst it is equal to the mean of IT values when diffuse severe changes are present (HHF case, right).

Also Mean and Median values of the two indexes provide distinct information, mean values being more representative of mild localized rather than of severe and diffuse atherosclerotic changes: different distribution patterns of lesions are present along each coronary artery, related to single lesion severity and extent of coronary involvement. But generally, mean and median values tend to coincide when lesions are present in all examined segments, whilst they diverge when atherosclerotic changes are localized only in few segments, such as in the proximal portion of main coronary arteries (Figure 1).

Independent variables selected by the model as associated to atherosclerotic burden and derived from implementations run on all diet treated cases are reported in Table 1 (lipoproteins and circulatory factors) and Table 2 (artery secreted proteins). As described in the Methods section, only variables with combined association of IT and LA histology indexes of atherosclerotic changes are reported and considered relevant.

**Table 1:** **Lipoproteins and inflammatory factors**.

	**Model selected circulatory variables**		
	**IT mean**	**IT median**	**LA mean**
IT median	ICAM-1 BAS, IL6 BAS		
LA mean	TNFα BAS	TNFα END-DIET	
LA median	OX-LDL END-DIET	HDL END-DIET	ICAM-1 END-DIET

Systemic inflammation selected variables: ICAM-1 (congruence with all indexes), TNFα (congruence with IT mean, IT median, LA mean). Lipoproteins selected: ox-LDL (congruence with IT mean, LA median), HDL end-diet (congruence with IT median, LA median).

**Table 2:** **Artery secreted proteins**.

	**Model selected proteins**		
	**IT mean**	**IT median**	**LA mean**
IT median	CATA, G3P, S10AA, CPNS1		
LA mean	CATA, CATD, BGH3, S10AA, CPNS1, ANXA4, FABPH	CATA, S10AA, CPNS1, PPCE	
LA median	CATA, BGH3, G3P, CATD, ANXA4	CATA, G3P, PPCE	BGH3, CATA, CATD, PPCE

Selected proteins: CATA (Catalase) is congruent with all indexes and combinations of histology indexes, CATD (Cathepsin D) with all indexes but not all combinations, S10AA (S100 A10) and CPNS1 (Calpain small subunit 1) with IT mean, IT median, LA mean, G3P (Glyceraldehyde-3-phosphate dehydrogenase) with IT mean, IT median, LA median, BGH3 (Transforming growth factor-beta-induced protein ig-h3) and ANXA4 (Annexin A4) with IT mean, LA mean, LA median, PPCE (Prolyl endopeptidase) with IT median, LA mean, LA median.

Among lipoproteins, oxLDL, and HDL are found significantly associated to arterial pathology. Plasma cytokine TNFα, as well as adhesion molecule ICAM-1 are also relevantly selected variables.

Among artery secreted proteins, the most selected and associated to all histology indexes of atherosclerotic burden are Catalase (CATA) and Cathepsin D (CATD). Transforming growth factor-beta-induced protein ig-h3(BGH3), S100A10 (S10AA) and Glyceraldehyde-3-phosphate dehydrogenase (G3P) are also selected and are congruent with 3 out of the 4 histology indexes.

### Selected variables by second implementation run of the model: two distinct groups (HF and HHF)

The model has been also applied to 8 weeks (HF) and 16 weeks (HHF) high cholesterol diet treated animals separately.

When considering systemic variables, separate analysis of early atherogenesis HF group does not provide further relevant information in addition to what previously derived from model run on pooled data: this is likely due to the limited number of HF cases and/or to the very low grade of atherogenesis after 8 weeks high cholesterol diet.

On the other hand, for local factors, the model selects Moesin and Osteonectine (MOES, SPRC) that had not been picked in the first run, as well as the already selected Apolipoprotein A4 (APOA4), Byglican (HPLN1), G3P, BGH3 and Calpastatin (ICAL), all related to lesion development in model run on HF and also on HHF group data. Annexin 1 (ANXA1) is the only protein selected from HF group omics data and unselected in HHF.

### Evaluation of regression coefficients in first and second implementation

Regression coefficients (absolute beta values, considering beta as the regression coefficients) of all the selected independent variables have been analyzed as an index of robustness of results and a qualitative measure of their relation with dependent variables IT and LA (mean and/or median values).

**Figure 2:**
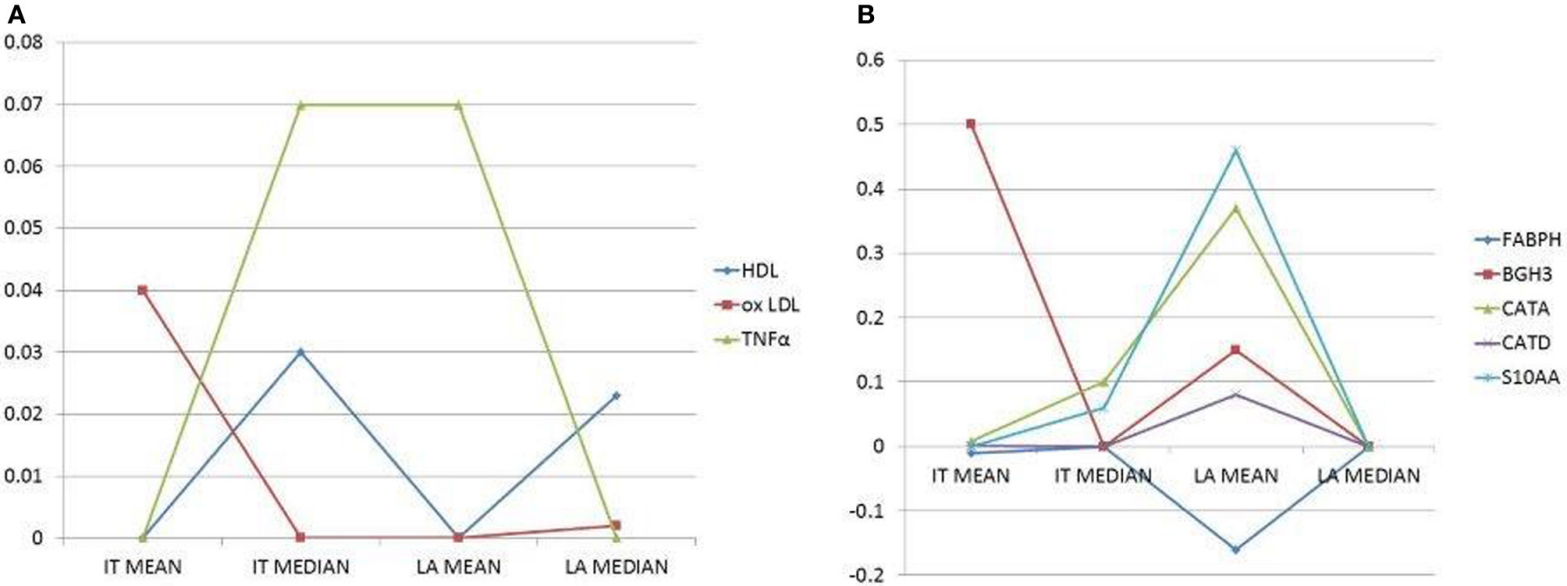
**(A)** Plots of selected circulatory variables (as shown by different symbols) with their coefficients (Y-axis). **(B)** Plots of selected, secreted proteins (as shown by different symbols) with their coefficients (Y-axis). Histomorphometry data on the X-axis are the dependent variables. Only independent variables (circulatory factors or secreted proteins) with the combined highest coefficients and congruent signs (i.e., inverse, negative sign, or direct, positive sign, correlations), for at least two dependent variables, are chosen and reported.

In pooled case run (first implementation), analysis of congruence by regression coefficients confirms systemic lipid and proinflammatory variables associated to atherosclerotic burden (association with at least one IT plus one LA index). A positive association is present for oxLDL, HDL and TNFα. On the other side, when considering artery specific factor congruence is limited to the combination of one IT and one LA index for CATA (positive association), CATD (positive), BGH3 (positive), S10AA (positive) and for Fatty acid-binding protein 3 (FABPH, with a negative association) (Figure 2).

Inclusion of regression coefficients, in variables selected by the model from separate run on HF and HHF groups, strongly restricts the relevance of results. No congruence is present, neither in absolute values nor in the sign of coefficients, whatever combination of dependent variables (histologic indexes) is considered.

## Discussion

The aim of this study is to propose a systems biology oriented approach as a tool to associate circulatory and tissue markers with coronary lesion development in a high cholesterol diet swine model. Using animal models, systemic and tissue data can be collected and analyzed at the early stages of diet-accelerated process and can be useful to define the timing of events that are mostly uninvestigable in the clinical setting.

Among animal models of atherogenesis, pig is currently considered the most suitable among those closer to human pathology (Vilahur et al., 2011).

Coronary histology indexes, plasma lipoproteins, circulatory cytokines, adhesion molecules and coronary specific secreted proteins were provided to the mathematical model and were chosen considering the current knowledge on factors involved in atherogenesis (Libby et al., 2002; Mohler et al., 2008).

The rationale of exploiting systems approaches through statistical models to elucidate the association between all these factors originates from the need of pointing out relationships strongly associated to coronary early atherosclerotic changes. It is known that the interplay between circulatory and tissue markers and the association between molecular factors and plaque growth represent the crossroad of blood-artery wall events during atherogenesis (Döring et al., 2012). Computational tools like those described, which run on a multitude of variables simultaneously, perform variable selection and model optimization, may help toward the ultimate aim of predictive inference, without bringing the burden of noisy and spurious correlative associations.

### BMA approach to experimental data

The model application to the provided data sets, followed by a post-processing exclusion based on the criteria of absolute values and sign of correlation coefficients, has evidenced that no congruence of any of the independent variables considered for all the chosen histology indexes is present, neither in the pooled nor in the separate HF and HHF data implementation runs. This finding is not surprising and underlies the limitations of this approach for pathophysiologic investigations when a reduced number of data is provided to the statistical tool. Despite such limitation, a restricted number of variables (two lipoproteins, one cytokine and five proteins) is finally suggested as robustly associated to both IT and LA morphologic indexes of atherogenesis outcome when HF and HHF cases are pooled. Separate analysis of the two groups does not lead to a robust selection of any variable under the criteria adopted, possibly because of the further reduction of data available for the model.

### Biological relevance of model results

The most robust association between dependent (histology indexes of atherosclerotic burden) and independent (circulatory and local factors) variables has been found when considering HF and HHF cases as a single group. This finding may be the consequence of model limitations (low number of early atherogenesis HF cases) although common mechanisms of initiation and of early plaque growth can also be hypothesized.

Circulatory associated variables are LDL, oxLDL, and TNFα and artery-specific variables are CATA, CATD, S100-A10, BGH3, and FABPH. It must be emphasized that, at variance with conventional statistical tools, the BMA mathematical model accounts for all the possible associations and blood-tissue factor interrelations in selecting those relevant for histologically determined atherogenesis outcome.

BMA selection of circulatory variables supports the current view that atherogenesis in a high cholesterol diet experimental model is related to systemic proinflammatory cytokines and adhesion molecules under a LDL-rich blood environment. The impact of inflammation-immunity state on pathology outcome has been demonstrated by several previous experimental and clinical studies, both as a strong proatherogenic determinant of plaque initiation as well as of its progression and evolution (Lamon and Hajjar, 2008; Merched et al., 2008).

By the mathematical model, identified local artery-specific factors, relevantly associated to lesion initiation and growth, are those mainly involved in macrophage/phagocytosis function and immunity-inflammatory pathways (CATA, CATD, S100-A10, FABPH, BGH3) (Haidar et al., 2006; Nacu et al., 2008; O'Connell et al., 2010; Lee et al., 2013). These proteins may be viewed as mediators and possible markers of a local inflammation scenario with pro- and anti-inflammatory elements playing a role in both initiation and early growth of high cholesterol diet-induced coronary atherosclerotic lesions. Among those, negative association is evidenced only for FABPH, in contrast with current knowledge on the role of this protein in atherogenesis (Lee et al., 2013).

## Concluding remarks

An integrative systems approach is proposed to study the association between circulatory markers and omics data to coronary atherosclerosis severity. BMA variable selection was chosen as a method to identify relevant factors associated to atherosclerosis. Specifically, TNFα was identified as an associated plasma marker, oxLDL and HDL were confirmed as relevant lipoproteins, macrophage related antioxidant Catalse enzyme, lysosome associated Cathepsin D, S100-A10 and Transforming growth factor-beta-induced protein ig-h3 were selected as associated to atherogenesis outcome.

The proposed approach has been shown to be feasible from a computational standpoint and capable of helping in understanding the association of multilevel factors in atherosclerotic plaque initiation with early growth.

The results of this study suggest a relevant conclusion: in a high-cholesterol diet-induced model of coronary artery disease, systemic inflammation impacts on atherogenesis outcome and it is specifically reflected by macrophage/phagocytosis-related artery-specific protein expression. Further studies integrating genomics, epigenomics and transcriptomics are needed for a better assessment of causative mechanisms and sequence of events in the early phase of atherogenesis in coronary artery disease.

### Conflict of interest statement

The authors declare that the research was conducted in the absence of any commercial or financial relationships that could be construed as a potential conflict of interest.

## Supplementary material

Supplementary Table 1 **Expression data for secretome proteins and circulatory factors in CNTL, HF and HHF groups**.

## References

[B1] Döring Y. Noels H. Weber C. (2012). The use of high-throughput technologies to investigate vascular inflammation and atherosclerosis. Arterioscler. Thromb. Vasc. Biol. 32, 182–195 10.1161/ATVBAHA.111.232686 22258901

[B2] Friedewald W. T. Levi R. I. Fredrickson D. S. (1972). Estimation of the concentration of low density lipoproteins cholesterol in plasma without use of the ultracentrifuge. Clin. Chem. 18, 499–502 4337382

[B3] Haidar B. Kiss R. S. Sarov-Blat L. Brunet R. Harder C. McPherson R. (2006). Cathepsin D, a lysosomal protease, regulates ABCA1-mediated lipid efflux. J. Biol. Chem. 281, 39971–39981 10.1074/jbc.M605095200 17032648

[B4] Lamon B. D. Hajjar D. P. (2008). Inflammation at the molecular interface of atherogenesis: an anthropological journey. Am. J. Pathol. 173, 1253–1264 10.2353/ajpath.2008.080442 18948435 PMC2570117

[B5] Leamer E. (1978). Specification Searches: Ad Hoc Inference with Non-Experimental Data. New York, NY: Wiley

[B6] Lee K. Santibanez-Koref M. Polvikoski T. Birchall D. Mendelow A. D. Keavney B. (2013). Increased expression of fatty acid binding protein 4 and leptin in resident macrophages characterises atherosclerotic plaque rupture. Atherosclerosis 226, 74–81 10.1016/j.atherosclerosis.2012.09.037 23122912 PMC3566542

[B7] Libby P. (2002). Inflammation in atherosclerosis. Nature 420, 868–874 10.1038/nature01323 12490960

[B8] Libby P. Di Carli M. Weissleder R. (2010). The vascular biology of atherosclerosis and imaging targets. J. Nucl. Med. 51 Suppl. 1, 33S–37S 10.2967/jnumed.109.069633 20395349

[B9] Libby P. Ridker P. M. Maseri A. (2002). Inflammation and atherosclerosis. Circulation 105, 1135–1143 10.1161/hc0902.104353 11877368

[B10] Merched A. J. Ko K. Gotlinger K. H. Serhan C. N. Chan L. (2008). Atherosclerosis: evidence for impairment of resolution of vascular inflammation governed by specific lipid mediators. FASEB J. 22, 3595–3606 10.1096/fj.08-112201 18559988 PMC2537438

[B11] Mohler E. R. Sarov-Blat L. Shi Y. Hamamdzic D. Zalewski A. Macphee C. (2008). Site-specific atherogenic gene expression correlates with subsequent variable lesion development in coronary and peripheral vasculature. Arterioscler. Thromb. Vasc. Biol. 28, 850–855 10.1161/ATVBAHA.107.154534 18276914

[B12] Nacu N. Luzina I. G. Highsmith K. Lockatell V. Pochetuhen K. Cooper Z. A. (2008). Macrophages produce TGF-β-induced (β-ig-h3) following ingestion of apoptotic cells and regulate MMP14 levels and collagen turnover in fibroblasts. J. Immunol. 180, 5036–5044 18354229 10.4049/jimmunol.180.7.5036 PMC2847349

[B13] O'Connell P. A. Surette A. P. Liwski R. S. (2010). S100A10 regulates plasminogen dependent macrophage invasion. Blood 116, 1136–1146 10.1182/blood-2010-01-264754 20424186

[B14] Raftery A. E. (1995). Bayesian model selection in social research. Sociol. Methodol. 25, 111–163 10.2307/271063

[B15] Raftery A. Hoeting J. Volinsky C. Painter I. Yeung K. Y. (2010). BMA: Bayesian Model Averaging. R package version 3.13. Available online at: http://CRAN.R-project.org/package=BMA

[B16] Rocchiccioli S. Pelosi G. Rosini S. Marconi M. Viglione F. Citti L. (2013). Secreted proteins from carotid endarterectomy: an untargeted approach to disclose molecular clues of plaque progression. J. Trasl. Med. 11, 260 10.1186/1479-5876-11-260 24131807 PMC3853772

[B17] Viglione F. Sbrana S. Puntoni M. Rocchiccioli S. Cecchettini A. Trivella M. G. (2013). Circulatory inflammation molecules and extracellular matrix proteoglycans: local and systemic modulated markers in an atherogenesis model. Eur. Heart J. 34 Suppl., 443 10.1093/eurheartj/eht308.P239922942340

[B18] Vilahur G. Padro T. Badimon L. (2011). Atherosclerosis and thrombosis: insights from large animal models. J. Biomed. Biotechnol. 2011:907575 10.1155/2011/907575 21274431 PMC3022266

[B19] Weber C. Noels H. (2011). Atherosclerosis: current pathogenesis and therapeutic options. Nature 17, 1410–1422 10.1038/nm.2538 22064431

