# Supplementary material for: Inflammation blood and tissue factors of plaque growth in an experimental model evidenced by a systems approach
Source: Front Genet. 2014 Apr 7;5:70. doi: 10.3389/fgene.2014.00070 (PMC3985011; doi:10.3389/fgene.2014.00070)
Supplement: Supplementary file 1 [file DataSheet1.DOCX]

The dependent variables (tagged by *histo_mean, histo_median, lam*) are regressed

over the independent variables, reported below by group:

- **7 by 10 tissue comp. + inflammation**
- **4 by 10 lipoproteins**
- **6 by 10 systemic inflamm. markers**
- **39 by 10**  **secretome** - in this case, a selection of proteins from the initial set has been operated to eliminate imbalanced data

across the 10 available samples, i.e.

| **L3** | **L9** | **L10** | **L11** | **HHF1** | **HHF4** | **HHF6** | **HHF7** | **HHF8** | **HHF10** |
| --- | --- | --- | --- | --- | --- | --- | --- | --- | --- |

The BMA model entry is thus using the aggregate formed by such groups, say **AGGR_lN(56,10)**

With **_lN**, the indication is that the data have been log-transformed.

The data have then been normalized based on the averaged controls.

Example:

BMA_output for dependent variable *Histo_meanIT*

Package Call:

***iBMA.bicreg.matrix(x = t(AGGR_lN), Y = first_DepVar_lN[1, 1:10])***

**iBMA:** shows an iterative operating mode of the algorithm,

***bicreg*** Bayesian Model Averaging for linear regression models.

Note:

BMA accounts for the model uncertainty inherent in the variable selection

problem by averaging over the best models in the model class according to approximate posterior model probability.

Separate effects were also accounted in the model computations, by splitting the samples into blocks (i.e. 1:4 and 5:10).
